# The progress of research into pseudophosphatases

**DOI:** 10.3389/fpubh.2022.965631

**Published:** 2022-08-29

**Authors:** Deqiang Liu, Yiming Zhang, Hui Fang, Jinxiang Yuan, Lizhen Ji

**Affiliations:** ^1^College of Life Sciences, Shandong Normal University, Jinan, China; ^2^The Collaborative Innovation Center, Jining Medical University, Jining, China

**Keywords:** pseudophosphatase, protein tyrosine phosphatases (PTPs), myotubularin phosphatases (MTMs), phosphatase and tensin homologues (PTENs), dual specificity phosphatases (DUSPs)

## Abstract

Pseudophosphatases are a class of phosphatases that mutate at the catalytically active site. They play important parts in many life processes and disorders, e.g., cell apoptosis, stress reaction, tumorigenesis, axon differentiation, Charcot-Marie-Tooth, and metabolic dysfunction. The present review considers the structures and action types of pseudophosphatases in four families, protein tyrosine phosphatases (PTPs), myotube protein phosphatases (MTMs), phosphatases and tensin homologues (PTENs) and dual specificity phosphatases (DUSPs), as well as their mechanisms in signaling and disease. We aimed to provide reference material for the research and treatment of related diseases.

## Introduction

Phosphatases catalyze the hydrolysis of phosphate monoester bonds, releasing phosphate ions and exposing free hydroxyl groups on protein substrates. On mutation of the catalytic active signature motif, HCX5R, or other catalytic domains, activity may be markedly reduced, converting the enzyme into a pseudophosphatase, accounting for about fourteen percent of the phosphatase family (1). The first pseudophosphatase was identified in the 1990s, when the Asp-181 mutant in PTP1B was found. This mutant produces an enzyme that competes with endogenous PTP1B for substrate, mainly promoting the accumulation of epidermal growth factor receptor (EGFR) and phosphate tyrosine on 120, 80 and 70 kDa proteins (2). With the discovery of additional pseudophosphatases, significant interest developed around their function, such as the regulation of mitogen activated protein kinase (MAPK) signaling, ubiquitylation, stress reaction, cell apoptosis, RhoA signaling and neuronal differentiation (3). However, once misregulation occurs, a disease state may arise. Nephrotic syndrome, chronic obstructive pulmonary disease (COPD), Charcot-Marie-Tooth (CMT) disorder, diabetes mellitus, obesity, leukocythemia, tumors (such as breast cancer, colorectal cancer, liver cancer and glioblastoma) have all been suggested to involve pseudophosphatase activity (3).

The families of protein tyrosine phosphatases (PTPs), myotubularin phosphatases (MTMs), phosphatase and tensin homologues (PTENs) and dual specificity phosphatases (DUSPs) are the most likely to have pseudophosphatase members. The catalytic activity of pseudophosphatase proceeds *via* four main modes (4): competitive binding with the substrate of the active phosphatase (5–7); binding to the phosphatase and modulating its activity (8–15); anchoring of the substrate in a specific subcellular localization (7, 12, 16); binding to proteins from intracellular signaling pathways to alter signal integration (6, 7, 17–27). Structural differences have been noted among pseudophosphatases, accounting for variations in their modes of action and function. The structural and functional characteristics of the four pseudophosphatase types will be discussed.

### Pseudophosphatases of the protein tyrosine phosphatase family

Four critical catalytic motifs characterize members of the PTP family: phosphate-binding (PTP) loop, phosphotyrosine recognition (KNRY) loop, catalytic site surface (WPD) loop and the catalytic-water motif (Q loop for short). Firstly, the PTP loop is composed of the catalytic active site motif, HCX5R or HCSAGXGR; there, cysteine can bind to the phosphate, while arginine stabilizes the phosphatase intermediate. Secondly, the KNRY loop occurs within a deep pocket for hydrolysis of serine or threonine phosphate. Thirdly, closure of the WPD loop is necessary for the hydrolysis of aspartic acid-catalyzed phosphates with acid-base catalysis by the aspartic acid residue. Fourthly, the Q loop can regulate the water molecules needed for the hydrolysis, avoids the phosphoenzyme intermediate as a kinase to phosphorylate unsuitable substrates and ensures phosphoryl transfer to the water (28). Numerous sequence variants have been generated within these high conservative motifs to define pseudophosphatases in the PTP family (4). Such pseudophosphatases mainly exist in the D2 domain of receptor-linked protein tyrosine phosphatases (RPTPs) and some non-receptor PTPs.

### D2 domain: Function of receptor-linked protein tyrosine phosphatases

Twelve human RPTPs are known, each consisting of a catalytically active membrane proximal D1 domain and a distal D2 pseudophosphatase domain. RPTPs may be divided into groups according to the characteristics of their extracellular domains: R1 refers to PTP receptor C (PTPRC/CD45); R2A to PTP receptor D (PTPRD), PTP receptor F (PTPRF) and PTP receptor S (PTPRS); R2B to PTP receptor K (PTPRK), PTP receptor M (PTPRM), PTP receptor T (PTPRT) and PTP receptor U (PTPRU); R4 to PTP receptor A (PTPRA) and PTP receptor E (PTPRE); R5 to PTP receptor G (PTPRG) and PTP receptor Z (PTPRZ).

The RPTP D2 domain regulates phosphatase activity and the downstream signaling pathway. It has been implicated in substrate recognition, regulation of D1 catalytic activity and redox sensing (4). The D2 domains of PTPRK and PTPRM dephosphorylate two substrates, cell junction-related protein (PARD3) and platelet affinity protein 3 (PKP3). Generation of chimeric proteins, with different combinations of the PTPRK and PTPRM D1 and D2 domains, has shown that the PTPRK-D2 domain recruits Afadin for dephosphorylation by PTPRM-D1 (5). The PTPRC D1 and D2 domains are predicted to form a metamorphic bag that inhibits receptor activity when bound to a small molecule (8). A principal effect of PTP oxidation is the inactivation of target proteins and the promotion of signaling by reactive oxygen species (ROS) (29, 30). ROS are continuously produced in humans and are associated with the pathogenesis of aging. It has been reported that the H_2_O_2_ (the most stable form of ROS) signaling pathway is selectively regulated by PTPRA in cells. Therefore, PTPRA may regulate aging through the ROS pathway (31). The PTPRA D2 domain is more sensitive to oxidation than the D1 domain *in vitro* (32). The redox-induced conformational changes in PTPRA led to the formation of rotational coupling and PTPRA dimer, which masks the extracellular hemagglutinin (HA) tag and transmits signals from the cytomembrane to the extracellular domain (17).

### Structural characteristics and functions of pseudophosphatases in non-receptor PTPs

Pseudophosphatases in non-receptor PTPs include non-receptor tyrosine protein kinase 23 (HD-PTP/PTPN23), non-receptor tyrosine protein kinase 14 (PTPN14) and non-receptor tyrosine protein kinase 21 (PTPN21). All have a single PTP domain that includes a complete HCX5R motif as well as the mutated KNRY and WPD loops (4).

#### PTPN23

PTPN23 contains an alanine to serine mutation, reducing its catalytic activity and generating a pseudophosphatase (33).

PTPN23 participates in physiological and biochemical processes including (1) inhibition of the pathogenesis of breast cancer and hepatocellular carcinoma (HCC) (18, 34) where PTPN23 inhibits endothelial cell migration through dephosphorylation of focal adhesion kinase (FAK) and interaction with Src kinases (9, 10). Loss of PTPN23 activates SRC and phosphorylation of the E-cadherin/β-catenin signaling complex, thereby enhancing cell motility and promoting the growth and diffusion of tumors (35). (2) PTPN23 may be involved in neonatal development; mice with homozygous knockout of PTPN23 suffer embryonic death (36). Heterozygous loss easily promotes the development of sporadic lung adenoma and B-cell lymphoma, as well as Myc-driven lymphoma in mice (37). It has been suggested that homozygotic loss of PTPN23 may cause embryonic or neonatal death in humans. (3) PTPN23 has a major role in neural development. The decline of PTPN23 function is associated with autosomal recessive syndromes causing hypoevolutism, abnormal brain structure (mainly encephalatrophy and/or ventriculomegaly), optic atrophy, intellectual disability, speech disorders, nanocephaly and epilepsy (38). (4) PTPN23 participates in the transportation of ubiquitin receptors by binding to the endosomal sorting complex required for transport (ESCRT) protein. PTPN23 can sort the endosomal cargo into intraluminal vesicles and form multivesicular bodies (MVBs), cooperatively with the ESCRT. The MVBs in turn fuse with the lysosomes, ensuring the efficient degradation of EGFR. PTPN23 depletion reduces the transfer of EGFR to lysosomes, causing the increase of ubiquitinated proteins in endosomal compartments and damaging MVB generation, preventing the deubiquitination of the receptor and lysosomal degradation, and further leading to sustained signal transduction and cell migration (39, 40). Combined effects on the Src family of kinases and ESCRT complexes reflect the role of PTPN23 in signal integration.

#### PTPN14

Unlike other active PTPs, human non-receptor PTPN14 lacks a KNRY ring and cannot recognize phosphotyrosine (33). As a result of its absence of catalytic activity, PTPN14 has been speculatively allocated to the pseudophosphatases group.

PTPN14 has tumor inhibitory effects. Investigations of p53 deficiency in pancreatic cancer have shown increased YAP signaling that indicates the mutual exclusivity of PTPN14 and TP53 mutations and the requirement of PTPN14 for p53-mediated tumor suppression in human cancers (41). Besides, PTPN14 restrains the survival, proliferation and invasion of pancreatic cancer cells by inhibiting YAP activity and reducing the TP53 mutation rate (41). Moreover, PTPN14 has a positive regulatory effect on the migration and invasion of gastric cancer cells; inhibition of PTPN14 can reduce the epithelial-mesenchymal transition in gastric cancer cells (42–44). In addition, knockout of PTPN14 promotes the growth and metastasis of breast cancer xenografts. Breast cancer cells that express PTPN14 lacking catalytic activity show increased secretion of growth factors and cytokines and increased EGFR expression (45). One clinically relevant study showed that PTPN14 can mediate the recovery of vascular endothelial-cadherin dephosphorylation and adhesion junction (AJ), thereby restoring endothelial barrier function. Therefore, PTPN14 may offer a therapeutic target for regulating endothelial barrier function and restoring stable AJ during lung inflammation and injury (46).

#### PTPN21

PTPN21 and PTPN14 have similar structures, PTPN21 having an RNRF motif in place of the PTPN14 RSRI motif. PTPN21 is mapped to the adhesion sites together with the actin filaments.

The following functions have been reported for PTNPT21: (1) PTPN21 shows tumor suppression activity and is highly expressed in bladder cancer tissue samples. Knockout of PTPN21 promotes EGFR degradation and inhibition of downstream ERK signaling, thereby reducing the growth and motility of bladder cancer cells (21). (2) PTPN21 binds to the stalk region of the Kinesin Family Member 1C (KIF1C) through its N-terminal FERM domain and stimulates dense core vesicle transport in primary hippocampal neurons, an action that is independent of phosphatase activity (47). (3) PTPN21 is also involved in homeostatic control in hematopoietic stem cells (HSCs). Knockout of PTPN21 in a mouse model increased the phosphorylation of Spetin1, impaired cytoskeletal remodeling, cortical instability, decreased cell rigidity and enhanced mobility, indicating the role of PTPN21 in dephosphorylation of the Tyr^246^ residue of Spetin1 (11). (4) PTPN21 forms a stable structure with actin, Src tyrosine kinase and FAK to modulate Src-FAK signaling and promote cell adhesion and migration, indicating its regulation of signaling pathways (22).

## Pseudophosphatases within the myotube protein phosphatase family

Six genes within the 14 members of the MTM family encode pseudophosphatases: MTMR5, MTMR9, MTMR10, MTMR11, MTMR12 and MTMR13 (1). These pseudophosphatases usually interact with active phosphatases to form dimers and perform specific functions.

MTMR5 can interact with the coiled-coil domain of MTMR2 to increase the latter's activity and determine subcellular localization (12). MTMR9 recruits MTMR6 and MTMR8 to the intermediate compartment and Golgi apparatus, forming dimers, MTMR6-MTMR9 and MTMR8-MTMR9, to regulate endoplasmic reticulum transport to the Golgi and to sustain Golgi integrity (13). A single nucleotide polymorphism of MTMR9 has been associated with obesity and hypertension. The transcript level of MTMR9 in the murine hypothalamic region is elevated after fasting and decreased after a high-fat diet, suggesting that MTMR9 may participate in the development of obesity and hypertension by regulating hypothalamic neuropeptides. Therefore, MTMR9 provides a candidate target for the development of new drugs for the prevention and treatment of obesity (48). MTMR10 mRNA is significantly decreased in achalasia, a rare disorder of esophageal peristalsis (49). The expression of MTMR11 would be significantly decreased in acute myeloid leukemia (50). Catalytically inactive MTMR12 binds skeletal muscle myotubularin. Disruption of this interaction decreases MTMR12 and myotube protein expression in skeletal muscle, and then leads to its defects and motor function damage. Thus, the interaction of myotube proteins with MTMR12 stabilizes the functional protein complex in skeletal muscle (51). MTMR12/3-PAP stimulates the relocation of myotube proteins from the plasma membrane to the cytosol, thereby altering the phenotype resulting from myotubularin overexpression (16). In conclusion, active and inactive MTM heterodimer formation is context dependent, regulates the subcellular localization of active phosphatases and enhances the stability of complexes with phosphatase activity.

Homodimer formation among both active and inactive MTMs has also been reported (4, 52, 53). For example, MTMR13/SBF2 and MTMR2 form homodimers via the coiled coil domain and then combine to form tetramers. Some undimerized MTMs, comprise a pseudophosphatase (e.g., MTM15/FAN) and an active phosphatase (e.g., MTM14), can interact directly (52). Either the lipid phosphatase MTMR2 or its regulatory binding partner, MTMR13/SBF2, has been related to the demyelinating peripheral neuropathy CMT disease type 4B (CMT4B) (14). MTMR2 normally functions to dephosphorylate PtdIns3P and PtdIns (3,5) P2, forming phosphatidylinositol and PI-5-P, which regulate endosome-lysosomal transport (14). Overexpression of MTMR2 leads to reduced AKT activation due to EGFR degradation. However, MTMR13 and MTMR2 are antagonistic within their dimeric complex leading to EGFR degradation, sustained AKT activation and regulation of membrane trafficking by MTMR2.

## Pseudophosphatases within the tensin protein family

The cytoplasmic phosphoprotein, tensin, is concerned with focal adhesions at the junction between cells and the extra-cellular stroma (23). Tensin includes a phosphotyrosine-binding (PTB) domain and an Src Homology 2 domain capable of interacting with tyrosine-phosphorylated proteins from its actin-bound localization. Such interactions allow the linkage of the intracellular signaling system with the cytoskeleton (23). Of the 4 members of the tensin family, tensins 1 and 2 are pseudophosphatases (23, 54).

### Structure and function of tensin 1

Tensin 1 is a cytoplasmic phosphoprotein containing 220 kDa that links the extracellular matrix (ECM), actin cytoskeleton and signal transduction (23, 54, 55). Its sequence contains Cys to Asp mutations, making it a pseudophosphatase.

The tensin 1 has been implicated in mammary cancer occurrence and development. Tensin 1 knockout leads to the excessive activation of cell division cycle 42 (cdc42), promoting invasion and metastasis (56) while its overexpression inhibits RhoGTPAase cdc42 activation, promoting invasion and metastasis (57). Maintenance of appropriate pseudophosphatase balance is thus suggested to be significant for normal breast physiology. In addition, highly expressed tensin 1 is also found in human colorectal cancer (CRC). Through the analysis of transgelin and its downstream target, tensin1, the mRNA and protein levels were found to be significantly increased in CRC patients; inhibition of transgelin or tensin1 reduced the proliferation and invasion of tumor cells. This indicates that tensin 1 may be involved in the development of CRC through transgelin/tensin1 signaling and that transgelin/tensin1 might serve as a prognostic and therapeutic target in CRC (54). Furthermore, tensin 1 is found to be involved in muscle activity in COPD. Increased expression of tensin 1 in the airways of COPD patients may promote airway obstruction by enhancing the expression of contractile proteins and their localization to stress fibers in human airway smooth muscle cells (58).

### Tensin 2

Tensin 2 has a His to Tyr mutation and lacks arginine, making it a pseudophosphatase. The protein is the main component of focal adhesions and shows high homology with tensin 1 in the terminal stress fiber. Unlike other tensin proteins, the N-terminal region of tensin 2 contains a C1 region (54).

Tensin 2 plays a critical role in renal failure and liver cancer (24). It is expressed in the podocytes and renal tubular epithelial cells of the normal kidney but not in the kidneys of mice with congenital nephrotic syndrome (ICGN). ICGN mice express a tensin 2 molecule with an eight-nucleotide deletion, CACCTACT, and show podocyte damage, albuminuria and tissue edema. In addition, tensin 2 knockout mice exhibit glomerular filtration barrier dysfunction (59). The effect caused by tensin 2 deletion in this model illustrates the protein's key role in normal renal function and suggests that this pseudophosphatase may be involved in renal failure. Tensin 2 interacts with tumor suppressor, DLC1, through its PTB domain, forming a caveolin 1-binding complex through the caveolin 1 binding motifs present on both proteins. The *DLC1* gene encodes a Rho GTPase activator protein domain and exhibits growth inhibitory activity in HCC cell lines. When tensin-2 complexes with DLC1 on the caveolae, it inhibits hepatoma cell growth (60).

## Pseudophosphatases within the DUSP family

DUSPs are classical cysteine-based protein phosphatases capable of dephosphorylating both phosphorylated serine/threonine and phosphorylated tyrosine residues (61). The DUSP family includes three pseudophosphatases: phosphoserine/threonine/tyrosine-interacting protein (STYX), STYX-like-1 (STYXL1) and STYX-like-2 (STYXL2). All contain glycine (G), isoleucine (I), serine (S) and arginine (R) at the active site (1).

### Structure and function of STYX

STYX contains a cysteine to glycine (HGX5R) mutation which renders it inactive. Its normal activity allows it to hydrolyze p-nitrophenyl phosphate and dephosphorylate the Tyr (P) and Thr (P) residues of MAPK homologous peptide sequences (62). If glycine is reverse mutated to cysteine, the catalytic activity of STYX can be restored, allowing it to dephosphorylate recombinant phospho-ERK (6).

STYX is involved in regulating the ERK signaling pathway. Styx locates in the nucleus, competes with DUSP4 for binding to mitogen activated protein kinase ERK, and then anchors ERK1/2 in the nucleus to regulate ERK1/2 localization and signal transduction (6, 63). Reduced STYX increases intracellular ERK activity, resulting in ERK-dependent fragmentation of Golgi complex, inhibiting Golgi polarization and directional cell migration. Over-expression of STYX decreases ERK1/2 activity, blocking the differentiation of PC12 cells (6). STYX affects sperm development by associating with mRNA binding protein, CRHSP-24 (a 24 kDa calcium responsive thermostable protein). Ablation of STYX expression disrupts the development of round and elongating spermatids and reduces sperm production (7). STYX is involved in cancer development through its regulation of signals for ubiquitination. Overexpression of STYX and its binding to FBXW7, regulates the ubiquitination of the ubiquitin ligase SCF complex, promotes proliferation, migration, invasion and the epithelial-mesenchymal transition of colorectal cancer cells (24). STYX also suppresses apoptosis and promotes carcinogenesis by inhibiting FBXW7 expression in endometrial carcinoma and promotes cell proliferation through the Notch-mTOR signaling pathway (25).

### Structure and function of STYXL1

STYXL1 is a member of the mitogen-activated protein kinase (MKP) subfamily but lacks the critical histidine and nucleophilic cysteine residues in its active site, rendering it catalytically inactive. It is also known as MK-STYX (64).

MK-STYX binds to mitochondrial phosphatase 1 (PTPMT1), reducing its phosphatase activity and the cardiolipin content of cells which compromises mitochondrial cristae morphology, reducing the efficiency of the electron transport chain and promoting apoptosis (65). MK-STYX also plays an important part in the stress reaction when it combines with G3BP-1 (the nucleation factor of stress particles) inhibiting G3BP-1-induced and sodium arsenite-induced stress granules. Mutation of phenylalanine and serine to histidine and cysteine within the MK-STYX active site signature motif generates the active phosphatase. Mutant MK-STYX phosphorylates G3BP1 resulting in the production of stress granules, the opposite effect to that produced by native MK-STYX (15). Over-expression of MK-STYX decreased activation of the Ras homologous gene family member A (RhoA), inhibiting downstream targets (e.g., cofilin), and promoting the conversion of actin monomers into polymers to induce neurite-like outgrowths. MK-STYX changes the morphology of hippocampal primary neurons and neuronal over-expression of MK-STYX led to numbers of primary neurites exceeding those of normal neurons, increasing synapse formation (66). Wu found upregulation of MK-STYX in HCC, along with downregulation of the RNA-binding protein, CELF2, through the PI3K/Akt pathway which accelerated HCC proliferation and inhibited apoptosis, thereby promoting the malignant progression of HCC (67). STYXL1 can be significantly upregulated in glioblastoma (GBM). Silencing of STYXL1 can inhibit glioma cell growth, the formation and migration of soft agar colonies, as well as the growth of xenograft tumors. Therefore, inhibition of STYXL1 expression may be useful in the treatment of GBM patients with STYXL1 disorder (26).

### Structure and function of STYXL2

The *STYXL2/DUSP27* gene is located on human chromosome 1 and encodes an 1,158 amino acid protein. Its high expression is observed in both skeletal and cardiac muscle (68).

*DUSP27* is highly conserved among vertebrates, including zebrafish. Kandice et al. (69) evaluated the role of *DUSP27* in zebrafish muscle cells. When *DUSP27* expression was disrupted by insertion of a transgenic sequence, the result was impairment of movement. The *DUSP27* mutant showed near complete paralysis at the embryonic stage, generating very low levels of spontaneous winding movement and touch reaction. Knockout of *DUSP27* can not stop mitosis but leads to serious disorder of the contractile apparatus in muscle fibers. Therefore, *DUSP27* has been speculatively associated with muscle fiber contraction in humans. Besides, *DUSP27* localizes to the cytoplasm retaining a trace nuclear expression in C2C12 myoblasts, and its knockout inhibits myogenic differentiation. And further study suggests that *DUSP27* can work as a downstream target of the JAK1-STAT1-STAT3 pathway, which plays a vital part in cell multiplication and differentiation of the C2C12 myoblasts (68).

## Summary and outlook

Pseudophosphatases participate in many life processes. Further exploration of pseudophosphatase mechanisms will illuminate their roles in the disease state and contribute to clinical research and disease treatment.

Current studies have mainly focused on laboratory investigations of the relationship between pseudophosphatases and disease; clinically relevant methods of pseudophosphatase detection do not yet exist. However, colorimetric determination of phosphatase has been widely used in clinical practice for the adjunctive examination of obstructive jaundice, primary liver cancer, secondary liver cancer, cholestatic hepatitis and prostate cancer, suggesting that the clinical detection of pseudophosphatases could be readily developed. Pseudophosphatases are involved in GBM, gastric cancer, CRC, breast cancer, renal failure, obesity and many other diseases, indicating that they may offer important therapeutic targets for these diseases.

## Author contributions

LJ and JY conceptualized this research, provided resources, and helped to revise the manuscript. DL wrote the first draft of the manuscript and was responsible for reviewing and editing. YZ and HF helped to write and edit the manuscript. All authors contributed to the article and approved the submitted version.

## Funding

The authors would like to acknowledge the Natural Science Foundation of Shandong Province of China (Grant Number: ZR2018BC011, LJ), the Research Startup Fund of Shandong Normal University (Reference: 0111018, LJ) and the Research Startup Fund of Jining Medical University (Reference: 600791001, JY) for funding this project.

## Conflict of interest

The authors declare that the research was conducted in the absence of any commercial or financial relationships that could be construed as a potential conflict of interest.

## Publisher's note

All claims expressed in this article are solely those of the authors and do not necessarily represent those of their affiliated organizations, or those of the publisher, the editors and the reviewers. Any product that may be evaluated in this article, or claim that may be made by its manufacturer, is not guaranteed or endorsed by the publisher.
